# The relationships between anxiety, psychotic-like experiences and autism: a systematic review

**DOI:** 10.3389/fpsyg.2025.1549886

**Published:** 2025-10-13

**Authors:** Madeleine Rowe, Sukhi Shergill, Raka Maitra

**Affiliations:** ^1^Institute of Psychiatry, Psychology and Neuroscience, King's College London, London, United Kingdom; ^2^Kent and Medway Medical School, University of Kent, Canterbury, United Kingdom; ^3^Department of Brain Sciences, Imperial College London, London, United Kingdom

**Keywords:** psychosis, autism, risk, early intervention, anxiety

## Abstract

**Introduction:**

Research has suggested that anxiety may be responsible for the elevated levels of psychotic-like experiences (PLEs) seen in Autism. However, there has been no previous systematic review examining this relationship.

**Method:**

We conducted three separate searches of PubMed and Ovid (MEDLINE, PsycINFO, Global Health and EMBASE) until 31st June 2024 for articles reporting on the association between anxiety, Autism and PLEs. A total of 54 articles were reviewed, including research exploring the links between anxiety and PLEs (28 studies), Autism and PLEs (12 studies), Autism and anxiety (14 systematic reviews and meta-analyses).

**Results:**

Studies of Anxiety and PLEs: Thirteen reported a significant positive correlation, and ten studies indicated that individuals with anxiety were more likely to experience PLEs. Studies of Autism and PLEs: Seven identified a significant positive correlation, with three longitudinal studies reporting that autistic traits predicted PLEs. Studies of Autism and Anxiety: Seven reviews revealed increased prevalence rates of anxiety disorders among autistic individuals, while 9 identified potential mediators of this relationship, such as intolerance of uncertainty and IQ.

**Conclusions:**

These findings demonstrate the association of PLEs with Autism and anxiety disorders, suggesting that co-occurring Autism and anxiety may represent an at-risk group for psychosis. Such insights have important implications for psychosis prevention, indicating that anxiety intervention in autistic populations may reduce PLE incidence. However, application of these findings to autistic individuals is significantly limited by the lack of included studies utilizing clinical populations. Future research is needed to establish the causal role of anxiety in this relationship, particularly using clinical adult samples.

**Systematic review registration:**

https://www.crd.york.ac.uk/PROSPERO/view/CRD42024555930.

## 1 Introduction

At the start of the 20th century, psychiatrist Eugen Bleuler considered autism to be a central feature of schizophrenia (Crespi, 2010). Following Leo Kanner's conceptualization of autism as a childhood disorder, Autistic Spectrum Disorder (ASD) was redefined as a distinct condition, despite sharing both neurodevelopmental and genetic factors with psychosis (Hajdúk et al., 2023). Subsequent theorists have therefore posited that Autism and psychosis sit on a continuum, forming part of a spectrum of major mental illnesses with overlapping symptoms (Craddock and Owen, 2010). There is also evidence to suggest that the two are diametrically opposed conditions: reciprocal copy number variant loci have been identified that may predispose individuals to either disorder depending on whether they are deleted or duplicated (Kirov et al., 2009). Alternatively, the causation model proposes that while Autism may increase vulnerability to developing psychosis, the two ultimately remain separate conditions (Esterberg et al., 2008). Although there are a number of conflicting theories intending to explain the relationship between Autism and psychosis, there appears to be consensus that the association is not simply by chance (Chisholm et al., 2015).

Understanding the relationship between Autism and psychosis is essential, as the co-occurring conditions are linked with poorer clinical outcomes, including increased rates of depression and suicide (Upthegrove et al., 2018; Hedley and Uljarević, 2018). Furthermore, autistic individuals are more likely to experience psychosis than the general population, with reported prevalence rates of up to 34% (Ribolsi et al., 2022; Arciniegas, 2015). The reason behind this elevated risk is not well understood, however one theory is that anxiety may mediate the pathway between Autism and psychosis (Demetriou et al., 2018). Previous research has highlighted the role of anxiety in the development and maintenance of psychosis among neurotypical populations, particularly in relation to positive symptoms (Freeman and Garety, 2003; Monsonet et al., 2022). For example, a longitudinal study found anxiety during adolescence to be predictive of psychosis at age 24 (Morales-Muñoz et al., 2022). Additionally, anxiety was reported to be associated with increased severity of hallucinations and delusions (Hartley et al., 2013). A recent meta-analysis identified that 29% of individuals with first-episode psychosis (FEP) experienced a co-occurring anxiety disorder (Wilson et al., 2020). This notable relationship may reflect the similar cognitive processes involved in the two conditions, such as persecutory thoughts and anticipating threat, which may increase vulnerability to developing psychosis when experiencing significant anxiety (Freeman, 2007). This is especially important as previous research has consistently reported high rates of anxiety disorders within autistic populations, ranging from 20 to 82% (Skokauskas and Gallagher, 2009; Hollocks et al., 2019; Bougeard et al., 2021). Moreover, co-occurring Autism and anxiety has been linked with poorer social relationships and increased likelihood of bullying, which are further risk factors for psychosis (Ambrose et al., 2021; Maiano et al., 2016). Notably, a recent study reported that 51.4% of participants with first-episode psychosis had experienced bullying during their lifetime (Kosteletos et al., 2024). This highlights the importance of considering environmental stressors, such as traumatic experiences, when discussing elevated rates of psychosis in Autism (Sætren et al., 2024).

One way of gaining insight into the relationship between Autism and psychosis may be through exploring psychotic-like experiences (PLEs), referring to transient or brief disturbances that resemble symptoms of psychosis, experienced among the general population (Hinterbuchinger and Mossaheb, 2021). PLEs are associated with later development of psychosis, and are particularly useful to study due to their prevalence among the general population, enabling larger sample sizes than research involving individuals with a psychosis diagnosis (Yung et al., 2006). Applied instruments such as the Community Assessment of Psychic Experiences (CAPE) conceptualize PLEs as the experience of psychosis symptoms without the presence of a disorder, often reflecting lower frequency or distress (Stefanis et al., 2002). In contrast, the DSM-5 emphasizes that the symptoms of schizophrenia must be persistent and associated with marked functional impairment (Rahman and Lauriello, 2016; American Psychiatric Association, 2022). A systematic review and meta-analysis conducted by Kiyono et al. (2020) reported that PLE prevalence among autistic individuals ranged from 6 to 45%, depending on the subtype, which is significantly higher than rates seen in the general population. Similarly, research has indicated that anxiety symptoms may be associated with increased rates of PLEs, with common mechanisms such as hypothalamic-pituitary-adrenal (HPA) axis dysregulation linking the two conditions (Yamasaki et al., 2018). Despite this, there are no known previous systematic reviews exploring the relationship between Autism, anxiety and PLEs.

Furthermore, research into this field faces several challenges. Firstly, the wide variety of diagnostic criteria used for both Autism and PLEs has resulted in incredibly heterogeneous findings within the literature, making comparison difficult (Kiyono et al., 2020). Additionally, several measures of anxiety and PLEs have not yet been validated within autistic populations, casting doubt on the validity of the conclusions drawn as they may not accurately capture these constructs in Autism (Mingins et al., 2021). While this issue does not apply to research measuring autistic traits in non-clinical populations, the application of these findings to individuals with an Autism diagnosis must be considered as a further challenge (Lisøy et al., 2022b). Regardless of these limitations, understanding the relationship between Autism, anxiety and PLEs has the potential to improve our understanding of vulnerability to psychosis (Catone et al., 2017). Exploring this could contribute to better clinical outcomes and more targeted interventions for both autistic individuals and the broader population (Yung et al., 2006). For instance, screening for anxiety symptoms among autistic individuals, or for autistic traits among individuals with an anxiety disorder, could allow for the early identification of individuals at risk of psychosis. Moreover, PLEs are not only indicative of psychosis but also of a wide range of mental health conditions (Kiyono et al., 2020). Consequently, exploring potential explanations for their prevalence is essential.

This review will examine the individual associations between Autism, anxiety and PLEs, to seek insight into the relationship between all three variables, which represents a current gap in the literature. In order to understand the increased prevalence of PLEs among autistic individuals, this review endeavors to explore whether anxiety increases vulnerability to PLEs among autistic individuals. Due to the current paucity of research on this topic, three individual searches combining the three variables will be conducted to address the research question.

## 2 Method

This review was conducted following the Preferred Reporting Items for Systematic Reviews and Meta-Analyses (PRISMA) guidelines (Page et al., 2021). Prior to conducting our search, a review protocol was developed and registered on PROSPERO (registration number: CRD42024555930).

### 2.1 Literature search

Three separate searches were conducted to thoroughly address the research question: (1) Anxiety and PLEs, (2) Autism and PLEs, and (3) Autism and Anxiety. Multi-component reviews such as our current study have been described previously in response to complex research questions (Gough et al., 2012). Searches were conducted using the databases PubMed and Ovid (MEDLINE, PsycINFO, Global Health and EMBASE), from inception until 30th June 2024. Therefore, the timeframe of our searches extends beyond previous reviews conducted by Kiyono et al. (2020) and Hollocks et al. (2019). The search terms used were: (Anxiety OR Anxiety Disorder OR Generalized anxiety disorder OR Social anxiety disorder OR Panic disorder OR Phobia) AND (Psychotic-like experience^*^ OR Psychotic like experience^*^ OR PLE); (Autism OR Autism spectrum condition OR Autism spectrum disorder OR ASD OR Asperger's syndrome) AND (Psychotic-like experience^*^ OR Psychotic like experience^*^ OR PLE). Furthermore, as Autism and anxiety is a well-researched area we conducted an additional search restricted to previous systematic reviews and meta-analyses, using the terms: (Anxiety OR Anxiety Disorder OR Generalized anxiety disorder OR Social anxiety disorder OR Panic disorder OR Phobia) AND (Autism OR Autism spectrum condition OR Autism spectrum disorder OR ASD OR Asperger's syndrome).

### 2.2 Study selection

After removing duplicates, the titles and abstracts of potentially relevant studies were screened by one author against the inclusion and exclusion criteria, before being confirmed by an independent rater. For searches 1 and 2, the inclusion criteria were: (1) observational studies, including cohort, cross-sectional and case control studies, (2) reporting findings on the relationship between either Autism and PLEs or Anxiety and PLEs, and (3) published in English. Additionally, there were no restrictions on age or gender, and a formal diagnosis of Autism or an anxiety disorder was not required. The exclusion criteria were the following: (1) studies evaluating interventions or treatments for Autism, anxiety, or psychosis without relevant data on the relationship between these conditions, (2) studies with insufficient data or incomplete reporting that prevents assessment of the relationship between Autism, anxiety, and PLEs, and (3) studies involving subpopulations not relevant to the research question, such as family members. For search 3, the inclusion criteria were: (1) systematic reviews and meta-analyses, (2) reporting findings on the relationship between Autism and Anxiety, and (3) published in English. The exclusion criteria were: (1) reviews evaluating interventions or treatments for Autism or anxiety without relevant data on the relationship between these conditions, and (2) reviews involving subpopulations not relevant to the research question.

**Figure 1:**
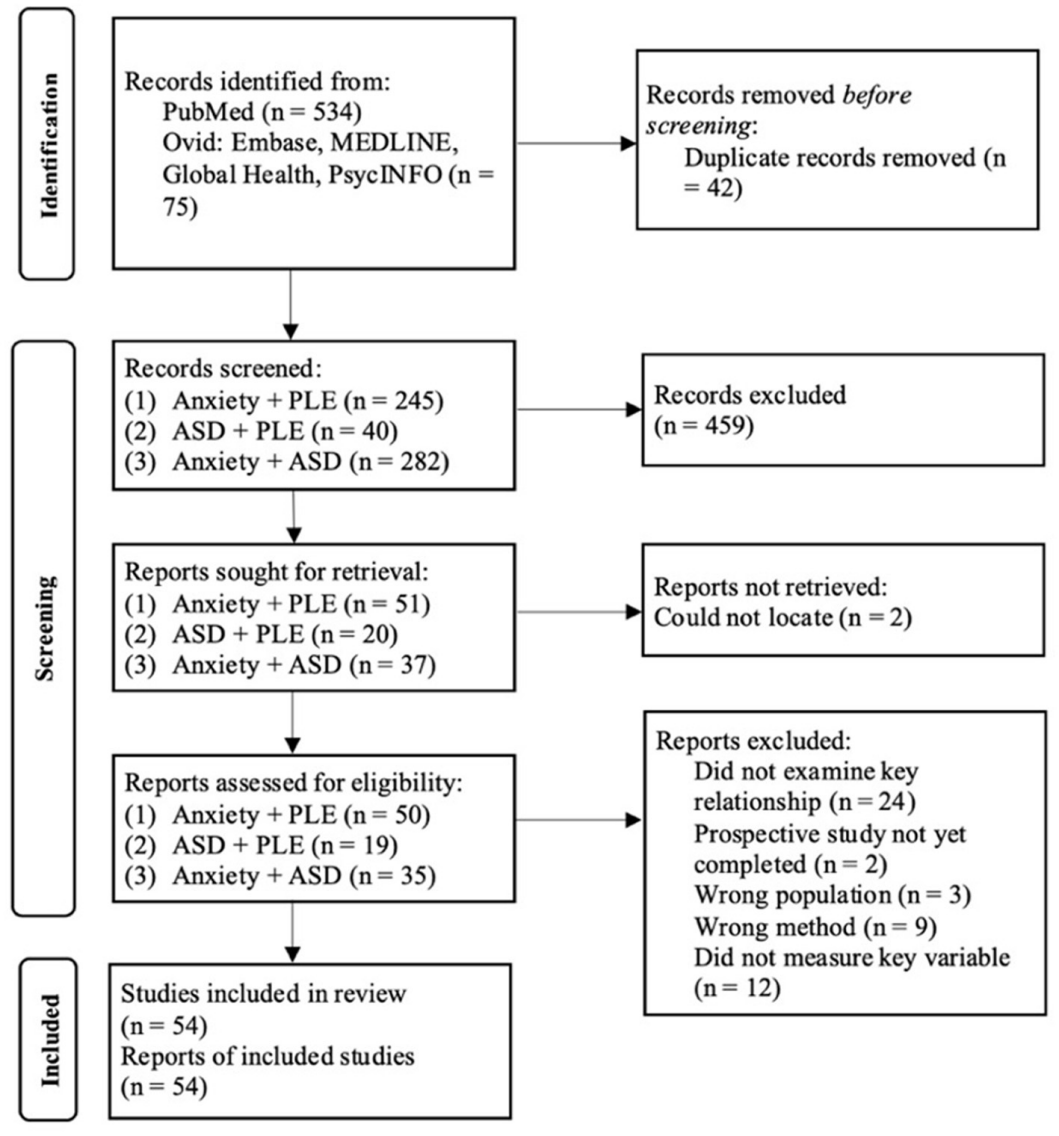
PRISMA flow diagram.

Studies that appeared to be eligible were retrieved for full-text assessment, conducted by one author and confirmed by an independent rater. Any uncertainty regarding study eligibility was resolved through discussion with a second author (see Supplementary material 1). Our initial search retrieved a total of 609 articles with 567 remaining after duplicates were removed. After titles and abstracts were screened, 459 articles were excluded leaving 108 for full-text evaluation. Following full-text evaluation, 54 articles met criteria for inclusion in this review. Articles were excluded for the following reasons: (1) they did not examine the relationship between the two key variables, (2) they involved the wrong population, (3) they used the wrong method, (4) they did not measure the key variable, (5) there was no full-text available and (6) it was a prospective study that had not yet been completed. The study selection process is summarized in the PRISMA flow diagram (see Figure 1).

### 2.3 Data extraction and quality assessment

The following variables of interest were extracted from the selected studies: author, year of study, study design, exclusion criteria, scales used (to measure anxiety, autistic traits or PLEs), and findings. Findings included means, standard deviations, effect sizes, *p*-values and correlation coefficients, where these measures were reported by the included studies. Microsoft Excel was used to create a data collection table and extraction was completed manually by one author. Quality of the included studies was assessed using the relevant Critical Appraisal Skills Programme (CASP) checklist. The CASP tool was selected as it provides checklists for both original research and previous systematic reviews and meta-analysis, allowing for a standardized assessment of quality to be used throughout (Ma et al., 2020). Studies were considered low quality if any of the first three questions were answered negatively, in accordance with previous guidance (Long et al., 2020).

### 2.4 Data synthesis and analysis

Due to the heterogeneity in study design and measures used among the included studies, a meta-analysis was not possible. Therefore, a narrative synthesis was conducted to address the research question.

## 3 Results

### 3.1 Study characteristics

#### 3.1.1 Anxiety and psychotic-like experiences

This search included a total of 28 studies published between 2011 and 2024. The studies were conducted across various settings: 22 were carried out in Western countries (USA, UK, Australia, France), while seven were conducted in non-Western countries (China, Japan, Turkey). The majority of the included studies utilized student populations and community samples, with a variety of methods including online surveys and in-person assessments. Many samples were drawn from non-clinical populations (*n* = 20), while eight involved clinical populations with diagnoses such as anxiety disorders, mood disorders, schizophrenia and psychosis. Most (*n* = 23) studies employed a cross-sectional design, while five studies utilized a longitudinal design with follow-up periods ranging from 3 to 21 years. One study combined both cross-sectional and longitudinal methodologies. The total sample size across the included studies was 127,564, with individual sample sizes ranging from 57 to 34,653. The average sample size across the studies was approximately 4,500 participants. Participants' age ranged from 9 to 73 years old (average age = 25), as the included studies involved both child and adult populations. Gender distribution was reported in 25 studies, with a general trend toward a higher proportion of females. Specifically, 15 studies had a majority female sample, while eight had a majority male sample. Two studies had an equal number of males and females.

A variety of validated instruments were used to assess anxiety and PLEs across the included studies. Anxiety was frequently measured using the State-Trait Anxiety Inventory (STAI) and the Beck Anxiety Inventory (BAI) in adult student and community samples, whereas child and adolescent populations utilized age-appropriate tools such as the Spence Children's Anxiety Scale (SCAS) and the Development and Well-Being Assessment (DAWBA). The Generalized Anxiety Disorder-7 (GAD-7) and Depression Anxiety Stress Scales (DASS) were also used in general population samples, particularly in online survey-based studies. In clinical samples or national epidemiological studies, structured diagnostic tools such as the Composite International Diagnostic Interview (CIDI) and Alcohol Use Disorder and Associated Disabilities Interview Schedule–DSM-IV (AUDADIS-IV) were employed, providing more rigorous diagnostic classification compared to self-report questionnaires.

PLEs were assessed using a similarly broad set of measures, with some variation depending on sample characteristics. The Community Assessment of Psychic Experiences (CAPE) was often used in student and community samples, reflecting its design to capture subclinical psychotic symptoms. The Prodromal Questionnaire (PQ) and Comprehensive Assessment of At-Risk Mental States (CAARMS) were more frequently used in studies involving clinical samples. In non-clinical samples, trait-like schizotypy were often assessed using instruments such as the Schizotypal Personality Questionnaire (SPQ), the Oxford-Liverpool Inventory of Feelings and Experiences (O-LIFE), and the Launay–Slade Hallucination Scale (LSHS). This variation in measurement tools across the included studies introduces notable heterogeneity; however, it also reflects appropriate methodological tailoring to the sample type.

#### 3.1.2 Autism and psychotic-like experiences

This search included 12 studies published between 2011 and 2024. The majority were conducted in a Western setting (USA, UK, Norway, Holland), with only two studies carried out in non-Western countries (Kenya, Hong Kong). Nine of the included studies used a cross-sectional design, two of which were case-control studies. Three were longitudinal studies, with follow-up periods ranging from 10 to 17 years. Most studies used stratified sampling methods within non-clinical populations, however those involving participants with an Autism diagnosis were recruited through local mental health services. Four studies utilized clinical populations. Of these, two involved participants with an Autism diagnosis, one included autistic individuals and individuals with an ADHD diagnosis, and the final study assessed autistic traits in participants with psychosis. Sample sizes across the studies ranged from 52 to 14,853 participants, with an average sample size of approximately 4,299 participants. Most studies focused on autistic traits in non-clinical populations, with only three involving individuals with an Autism diagnosis. Participants' ages ranged from 9 to 41 years (average age = 24). Of the included studies, 11 reported gender distribution, with seven having a majority female sample.

Autistic traits were measured in non-clinical populations using the following self-report measures: Autism Quotient (AQ), Social Communication Disorder Checklist (SCDC), Autism Symptom SElf-ReporT for Adolescents and Adults (ASSERT) and the Comprehensive Autistic Trait Inventory. Three studies involving clinical populations used the AQ, while the fourth did not include a trait measure and relied solely on a confirmed Autism diagnosis, reflecting a more categorical approach. PLEs were assessed using a range of instruments, including the CAPE, SPQ, PQ, Psychosis-Like Symptoms interview (PLIKSi), Washington Early Recognition Center Affectivity and Psychosis (pWERCAP) and the Psychosis Screening Questionnaire (PSQ). The Diagnostic Interview for Children (DISC-C) was utilized in one study involving younger participants (mean age = 12.6).

#### 3.1.3 Autism and anxiety

The final search included 14 systematic reviews and meta-analyses, published between 2011 and 2023. Specifically, six systematic reviews, four meta-analyses and four systematic reviews with meta-analyses. The number of studies included in each review ranged from 5 to 340. Moreover, the number of databases searched ranged from two to five, most commonly using PubMed, PsycINFO and Embase. Of the 14 reviews, 13 included populations with a primary Autism diagnosis and one involved individuals with a primary diagnosis of an anxiety disorder. Additionally, 10 reviews focused on children and adolescents, while four involved studies with adult participants. Three of the included reviews did not report conducting a quality assessment.

### 3.2 Quality assessment

The CASP checklists were used to assess methodological quality across studies. CASP assessment does not generate a cumulative score; instead, it provides a structured appraisal of key domains such as study design, bias and validity. This approach supports a nuanced evaluation of research strengths and limitations without oversimplifying quality into a numerical rating (Ma et al., 2020).

#### 3.2.1 Cross-sectional studies

Across the included cross-sectional studies, the majority were of moderate to high quality, with consistent reporting of clear aims and appropriate methodology. However, attention to confounding factors and clarity in effect estimation were variable, which may affect the strength of some conclusions. See Supplementary Table 1 for full CASP assessment of cross-sectional studies.

#### 3.2.2 Longitudinal studies

The included longitudinal studies were generally of high methodological quality. Most met all CASP criteria, with clear aims, robust measurement of exposures and outcomes, and well-described follow-up periods. Several studies fulfilled all or nearly all criteria, enhancing confidence in their findings. However, others demonstrated limitations in areas such as accounting for confounding factors or had incomplete follow-up which may impact the reliability of certain results. See Supplementary Table 2 for full CASP assessment of longitudinal studies.

#### 3.2.3 Systematic reviews

The included systematic reviews varied in methodological quality. Most had clearly defined aims, appropriate search strategies, and included relevant studies. However, several reviews showed limitations in critical areas such as quality assessment of included studies and reporting of cost-benefit considerations. While certain reviews demonstrated high methodological rigor across all criteria, others lacked transparency in combining results or assessing the validity of findings. These variations in quality suggest that conclusions drawn from the systematic reviews should be interpreted with caution, particularly when based on those with less thorough appraisal or reporting. See Supplementary Table 3 for full CASP assessment of systematic reviews.

#### 3.2.4 Systematic review and meta-analyses

All included systematic reviews with meta-analyses were of consistently high methodological quality. Each met the CASP criteria for clear focus, rigorous search strategies, inclusion of relevant studies, and thorough appraisal of validity and limitations. Notable reviews demonstrated comprehensive and transparent reporting, appropriate data synthesis, and thoughtful discussion of applicability and added value. The consistent strength of these reviews enhances confidence in the findings they report and their contribution to the overall synthesis. See Supplementary Table 4 for full CASP assessment of systematic review and meta-analyses.

### 3.3 Anxiety and psychotic-like experiences

From the total 54 studies, 28 reported findings on the relationship between anxiety and PLEs. Their characteristics and key findings are displayed in Table 1. Among these, 13 reported a significant positive correlation between anxiety and PLE scores. Ten studies reported odds ratios indicating that individuals with anxiety were more likely to experience PLEs. Conversely, three studies reported odds ratios suggesting that individuals experiencing PLEs were more likely to have an anxiety disorder. All five longitudinal studies reported that anxiety predicted PLEs at follow-up, or that PLEs predicted an anxiety disorder at follow-up. Four of the included studies explored potential mediators or factors involved in the relationship, including intolerance of uncertainty (IU) and other cognitive biases. Finally, five studies reported findings on specific anxiety disorders and PLEs, in addition to obsessive-compulsive disorder (OCD).

**Table 1:** Anxiety and psychotic-like experiences: characteristics and findings of studies meeting inclusion criteria (*n* = 28).

**Author (year)**	**Country**	**Study design sample (number and diagnosis)**	**Mean age (*SD*) gender ratio (M/F)**	**Outcome measures**	**Main findings and clinical implications**
Antosz-Rekucka and Prochwicz (2023)	Poland	Cross-sectional 108 non-clinical	20.73 (SD = 1.54) 108 F	State-Trait Anxiety Inventory (STAI) Community Assessment of Psychic Experiences (CAPE)	There was a positive correlation between anxiety scores and CAPE positive score (*r* = 0.54, *P* = 0.001). Additionally, there was a positive correlation between anxiety scores and CAPE negative score (*r* = 0.83, *P* = 0.001). These findings suggest that higher levels of anxiety may be associated with increased levels of PLEs, particularly negative PLEs such as social withdrawal and blunted affect.
Armando et al. (2013)	Italy	Cross-sectional 128 anxiety disorder 41 HC	21.1 (SD = 4.7) 21.8 (SD = 2.9) 44 M/125 F	Community Assessment of Psychic Experiences (CAPE) Beck Anxiety Inventory (BAI) Intolerance to Uncertainty Scale (IUS)	Individuals with social anxiety experienced significantly more positive [*F*(2) = 226, *P* < 0.001] and negative [*F*(2) = 43.38, *P* < 0.001] PLEs compared to healthy controls. Anxiety scores (BAI) were significantly correlated with both positive (*r* = 0.575, *P* = 0.001) and negative (*r* = 0.215, *P* = 0.05) CAPE scores. Additionally, PLEs were also associated with intolerance of uncertainty (*r* = 0.520, *P* = 0.001) among individuals with social anxiety. These findings indicate that a significant number of individuals with social anxiety may experience PLEs. Moreover, they indicate that anxiety increases as PLEs increase. This also supports the theory that affective distress may be involved in the formation of psychosis symptoms.
Baryshnikov et al. (2018)	Finland	Cross-sectional 282 with mood disorders	42.2 (SD = 13.1) 73 M/209 F	Community Assessment of Psychic Experiences (CAPE) Overall Anxiety Severity and Impairment Scale (OASIS) Schizotypal Personality Questionnaire-Brief form (SPQ-B)	There was a positive correlation between OASIS (anxiety) and CAPE scores (*r* = 0.64, *P* = 0.001). Most participants with a mood disorder (96.8%) reported experiencing PLEs at least ‘sometimes'. These findings indicate that anxiety may increase risk of PLEs among individuals with a mood disorder. Moreover, that there is an increased prevalence of PLEs among those with a mood disorder (bipolar disorder and major depressive disorder).
Bortolon and Raffard (2015)	France	Cross-sectional 36 OCD 49 schizophrenia 30 HC	37.72 (SD = 13.13) 31.26 (SD = 10.8) 32.33 (SD = 11.1) 71 M/44 F	The Launay–Slade Hallucination Scale (LSHS) Peters et al. Delusions Inventory (PDI-21) State-Trait Anxiety Inventory (STAI) Beck Depression Inventory II (BDI-II)	Individuals with OCD scored higher than healthy controls but lower than individuals with schizophrenia on the PDI [*F*(3, 111) = 6.884, *P* = 0.000]. The effect size was moderate (*ηp* 2 = 0.157). These findings suggest that individuals with anxiety have intermediate PLE scores, falling between controls and individuals with psychosis. They also provide support for the role of anxiety in the development of PLEs.
Bourgin et al. (2020)	USA	Cross-sectional 34,653 non-clinical	48.14 (SD = 15.67) 16,818 M/17,835 F	Alcohol Use Disorder and Associated Disabilities Interview Schedule–DSM-IV (AUDADIS-IV)	Lifetime prevalence of any anxiety disorder was 75% (SE = 1.18) for those who endorsed more than 5 PLEs. Participants with at least one PLE were more likely to have an anxiety disorder (OR = 2.96, 95% CI = 2.73–3.20) compared to those without any PLEs. This supports the association between PLEs and anxiety, especially the bidirectional relationship in which PLEs increase anxiety and anxiety increases PLEs.
Byrne et al. (2015)	Australia	Cross-sectional 1,823 non-clinical 82 anxiety disorder	73.28 (SD = 5.81) 904 M/1,001 F	Original questionnaire	Individuals with an anxiety disorder were significantly more likely to experience PLEs than those without (OR = 5.33, 95% CI = 1.97–14.42; *z* = 3.29, *P* = 0.001). These findings highlight the importance of screening for PLEs among older adults with anxiety disorders.
Deng et al. (2020)	USA	Cross-sectional 197 non-clinical	32.38 (SD = 10.7) 104 M/92 F	Oxford-Liverpool Inventory of Feelings and Experiences (O-LIFE) State-Trait Anxiety Inventory (STAI)	There was a significant correlation between PLEs and anxiety (*r* = 0.65, *P* = 0.001). The relationship between PLEs and (1) social and leisure activities (*z* = 6.50, *P* = 0.001), (2) extended family relationships (*z* = 6.50, *P* = 0.001) and (3) within family relationships (*z* = 3.79, *P* = 0.001) were mediated by anxiety. This further supports the relationship between PLEs and anxiety. Additionally, these findings suggest that anxiety interventions may reduce the impact of PLEs on domains of social functioning.
Ered et al. (2018)	USA	Cross-sectional 2,687 non-clinical	20.22 (SD = 3.21) 689 M/1,998 F	Prodromal Questionnaire (PQ) State-Trait Anxiety Inventory (STAI) Pittsburgh Sleep Quality Index (PSQI)	PLEs and anxiety were significantly correlated (*r* = 0.54, *P* = 0.01). Anxiety symptoms did not mediate the relationship between PLEs and sleep quality. These findings provide support for the association between PLEs and anxiety.
Freeman and Fowler (2009)	UK	Cross-sectional 200 non-clinical	37.5 (SD = 13.3) 100 M/100 F	Cardiff Anomalous Perceptions Scale Depression Anxiety Stress Scales (DASS)	Binary logistic regression found anxiety to be a significant predictor of PLE: anxiety predicted paranoia (*B* = 0.172, SE = 0.051, *P* = 0.001) and hallucinations (*B* = 0.073, SE = 0.034, *P* = 0.032). This provides evidence for the link between anxiety and paranoid thinking, including persecutory beliefs. The association with hallucinations is less clear and requires further investigation.
Giocondo et al. (2021)	Brazil	Longitudinal 1,712 non-clinical	9.7 (SD = 1.92) 53% M/47% F	Community Assessment of Psychic Experiences (CAPE) Comprehensive Assessment of At-Risk Mental States (CAARMS) Development and Well-Being Assessment (DAWBA)	CAPE scores (PLEs) at baseline predicted the likelihood of experiencing any anxiety disorder at 3-year follow-up (OR = 1.17, 95% CI 1.09 – 1.98, *P* = 0.05). Anxiety disorders at baseline predicted CAPE scores at 3-year follow-up (OR = 1.94, 95% CI = 1.04 – 1.89, *P* = 0.05). These findings support the bidirectional relationship between PLEs and anxiety, especially due to the longitudinal design.
Isaksson et al. (2020)	Sweden	Longitudinal 1,445 non-clinical	14.38 (SD = 1.04) 42% M/58% F	Unspecified PLE self-rating scale Spence Children's Anxiety Scale (SCAS)	Having PLEs was positively correlated with self-rated symptoms of anxiety at baseline (ρ = 0.385, *P* = 0.001) and three years later (ρ = 0.248, *P* = 0.001). Individuals with an anxiety disorder were more likely to experience PLEs at follow-up (OR = 1.11, 95% CI = 1.05–1.17, *P* = 0.001). This suggests that PLEs predict anxiety symptoms and conversely, anxiety symptoms predict PLEs.
Isaksson et al. (2022)	Sweden	Longitudinal 1,446 non-clinical	20.15 (SD = 1.08) 38% M/62% F	Unspecified PLE self-rating scale Spence Children's Anxiety Scale (SCAS)	Anxiety during adolescence predicted PLEs in adulthood (OR 1.02, 95% CI = 1.57–2.09, *P* = 0.010). Individuals with PLEs had significantly higher anxiety scores at baseline (*t* = 5.52, *P* = 0.001) and follow-up (*t* = 8.49, *P* = 0.001). Further support for the association between anxiety symptoms and PLEs, and their ability to predict each other.
Lu et al. (2022)	China	Cross-sectional 950 non-clinical	Not reported 950 F	Community Assessment of Psychic Experiences (CAPE) Generalized Anxiety Disorder Questionnaire (GAD-7)	The association between anxious symptoms and hallucinatory experiences was positive and significant (*r* = 0.126, *P* = 0.001). Anxiety was a risk factor for hallucinatory experiences (OR = 8.71, 95% CI = 2.12–3.80, *P* = 0.00). This study provides broader support for the association between anxiety and PLEs, within a sample of pregnant women.
Merola et al. (2024)	Italy	Cross-sectional 44 with psychosis 163 HC	29.64 (SD = 10.18) 49% M/51% F	Community Assessment of Psychic Experiences (CAPE) Symptom Checklist-90-revised (SCL-90-R)	Individuals with psychosis had significantly higher anxiety scores than healthy controls (*t* = 2.193, *P* = 0.029). Although, the effect size small (*d* = 0.373). PLE scores did not predict anxiety in the psychosis group (*t* = 0.458, *P* = 0.651), however they did in the healthy controls group (*t* = 6.97, *P* = 0.000). This may reflect that individuals with psychosis may be used to PLEs and subsequently are less distressed by them.
Monsonet et al. (2022)	Spain	Cross-sectional 65 high-schizotypy 74 ARMS 39 FEP	20.83 (SD = 1.96) 21.56 (SD = 4.02) 24.59 (4.88) 95 M/83 F	Experience sampling methodology	Anxiety significantly mediated the pathway from stress to PLEs, with an estimated effect size of 0.028 (SD = 0.005). Similarly, anxiety significantly mediated the pathway from stress to paranoia, and the estimated effect size was 0.032 (SD = 0.008). The similarity between anxiety and stress may have weakened the mediating effect, however it supports the role of anxiety in the development of PLEs and its role as a mediator.
Morales-Muñoz et al. (2022)	UK	Longitudinal 8,242 non-clinical	24 (SD = Not reported) 38% M/62% F	The Development and Well-Being Assessment (DAWBA) Psychosis-like Symptom Interview	High levels of anxiety throughout childhood and adolescence were significantly associated with PLEs at age 24 (OR = 2.02, 95% CI 1.26–3.23, *P* = 0.003). Persistent, high levels of anxiety were significantly associated with PLEs at age 24 (β = 0.028, *P* = 0.001). Furthermore, high anxiety was significantly associated with psychosis at age 24 (OR = 4.23, 95% CI 2.27–7.88, *P* = 0.001). These findings provide support for the role of anxiety in the development of PLEs, particularly due to the longitudinal methodology.
Park et al. (2022)	USA	Cross-sectional 57 non-clinical	20.38 (SD = 2.00) 25% M/75% F	Community Assessment of Psychic Experiences (CAPE) Beck Anxiety Inventory (BAI)	There was a positive correlation between PLEs and anxiety symptoms (*r* = 0.48, *P* = 0.01). There was also a significant interaction between PLEs and anxiety symptoms, *F*(3,44) = 4.35, *P* = 0.01. This suggests that while anxiety may contribute to PLEs, PLEs may also cause anxiety as they are a stressful experience.
Prochwicz and Gaweda (2016)	Poland	Cross-sectional 492 non-clinical	21.58 (SD = 2.48) 90% M/10% F	Community Assessment of Psychic Experiences (CAPE) State and Trait Anxiety Inventory (STAI)	There was a positive correlation between anxiety and positive (*r* = 0.16, *P* = 0.001) and negative (*r* = 0.26, *P* = 0.001) PLEs. These findings suggest that anxiety may have a stronger association with negative PLEs, although the difference is negligible.
Prochwicz and Kłosowska (2018)	Poland	Cross-sectional 170 non-clinical	24.01 (SD = 6.07) 15 M/153 F	Peters et al. Delusions Inventory (PDI) State and Trait Anxiety Inventory (STAI)	Trait anxiety significantly predicted PLEs (*B* = 0.30; β = 0.38, *P* = 0.001; *R*2 = 0.14). Among those with “high anxiety”, external attribution bias significantly predicted PLEs (*B* = 0.20; β = 0.23, *P* = 0.05; *R*2 = 0.05). Among those with “low anxiety”, attention to threat bias predicted PLEs (*B* = 0.29; β = 0.35, *P* = 0.05; *R*2 = 0.15). These findings support the association between negative emotions and PLEs.
Rejek and Misiak (2023)	Poland	Cross-sectional 1,100 non-clinical	27.1 (SD = 5.1) 49% M/51% F	Prodromal Questionnaire-16 (PQ-16) Generalized Anxiety Disorder-7 (GAD-7) Obsessional Compulsive Inventory-Revised (OCI-R)	Network analysis revealed strong positive association between OCD and PLEs, and a moderate positive association between GAD and PLEs. GAD had the greatest predictability for PLEs (0.637), and OCD had moderate predictability (0.503). These findings suggest a strong association with OCD, in line with previous literature. They also indicate that PLEs may be a marker of a wide range of psychopathologies.
Rep et al. (2023)	USA	Cross-sectional 33,510 non-clinical	Not reported	Alcohol Use Disorder and Associated Disabilities Interview Schedule–DSM-IV (AUDADIS-IV)	Across all age groups, individuals who reported PLEs displayed higher rates of any anxiety disorder: 20–29 year olds (OR = 5.07, CI = 4.28–6.02), 30–39 year olds (OR = 4.84, CI = 4.14–5.66), 40–49 year olds (OR = 4.64, CI = 4.06–5.30), 50–59 year olds (OR = 4.54, CI = 3.89–5.31), 60–69 year olds (OR = 4.44, CI = 3.64–5.42) and over 70 year olds (OR = 4.66, CI = 3.73–5.83). Individuals with PLEs were significantly more likely to have generalized anxiety disorders, especially in the 20–29 age group (OR = 16.06, CI = 11.13–23.19). These findings indicate that the association remains significant between anxiety and PLEs across age groups, although is strongest among those 20–29. Furthermore, having at least one PLE increased risk of an anxiety disorder, particularly GAD.
Saha et al. (2012)	Australia	Cross-sectional 580 anxiety disorder 10,061 non-clinical	Not reported	Composite International Diagnostic Interview (CIDI)	Individuals with anxiety disorders were significantly more likely to endorse both PLE screen (OR = 3.88, *P* = 0.001) and probe (OR = 3.36, *P* = 0.001) items. All anxiety disorders analyzed were significantly associated with PLEs: panic disorder (OR = 4.56), general anxiety (OR = 3.69), OCD (OR = 5.19), agoraphobia (OR = 5.18) and social phobia (OR = 4.14). These findings support the relationship between a range of anxiety disorders and PLEs. Also highlights the need for screening for PLEs among individuals with anxiety disorders.
Unterrassner et al. (2017)	Switzerland	Cross-sectional 206 non-clinical	33.11 (SD = 11.23) Not reported	The Revised Exceptional Experiences Questionnaire (PAGER) The Magical Ideation Scale (MIS) The Symptom Checklist-90-Revised (SCL-90-R)	All subscales of the PLE questionnaire were associated with subscales of the anxiety questionnaire, ranging from weak to moderate associations. Unusual perceptual experiences (*r* = 0.45, *P* = 0.000) and dissociative anomalous perceptions (*r* = 0.42, *P* = 0.000) were significantly associated with the general anxiety subscale. Ideas of reference was associated with the OCD subscale (*r* = 0.37, *P* = 0.000). This provides further insight into the relationship between subsections of PLEs and anxiety symptoms.
Varghese et al. (2011)	Australia	Cross-sectional and longitudinal 590 with anxiety 1,851 non-clinical	Not reported	Peters Delusional Inventory (PDI) The Composite International Diagnostic Interview (CIDI)	Individuals with an anxiety disorder reported significantly more PLEs when completing the PDI (OR = 5.81, 95% CI = 3.68–9.16). Those with anxiety disorders were significantly more likely to endorse any CIDI hallucination (OR 2.99, CI = 2.23–4.01) and delusion (OR = 2.60, CI = 1.95–3.46) item.
Xu et al. (2024)	China	Cross-sectional 18,578 non-clinical	20.07 (SD = 1.63) 32% M/68% F	Community Assessment of Psychic Experiences (CAPE) Generalized Anxiety Disorder (GAD-2)	Individuals with anxiety were more likely to experience frequent PLEs than those without (OR = 4.28, 95% CI = 1.03–1.94, *P* = 0.001). Anxiety was significantly associated with PLEs (*d* = 0.37, *P* = 0.001). These findings further highlight the association between anxiety and PLEs.
Yamasaki et al. (2018)	Japan	Longitudinal 887 non-clinical	14.0 (SD = 1.7) 50% M/50% F	General Health Questionnaire (GHQ-12) Diagnostic Interview Schedule for Children (DISC-C)	Anxiety significantly worsened over time among individuals who experienced PLEs (incidence) between the two time points (α1 = 1.91, *P* = 0.001). These results suggest that anxiety and PLEs may co-occur and provides longitudinal support for their association.
Yuan et al. (2024)	China	Cross-sectional 4,302 non-clinical	18.59 (SD = 0.95) 45% M/55% F	Community Assessment of Psychic Experiences (CAPE-P15) Generalized Anxiety Disorder-7 (GAD-7)	Individuals with anxiety symptoms were more likely to have experienced PLEs than those without (OR = 4.90, 95% CI = 3.10–6.43, *P* = 0.001). Additionally, individuals with PLEs had higher anxiety scores than those without PLEs (*t* = −30.95, *P* = 0.001). These findings indicate that PLEs may be an important marker for anxiety disorders.
Kafali et al. (2022)	Turkey	Cross-sectional 684 non-clinical	15.7 (SD = 3.43) 55% M/45% F	Community Assessment of Psychic Experiences (CAPE) Generalized Anxiety Disorder-7 (GAD-7)	Anxiety scores were significantly associated with scores on the positive CAPE subscale (*r* = 0.58, *P* = 0.001). Those with higher anxiety scores were more likely to experience PLEs than those without (OR = 1.2, 95% CI = 1.12–1.29, *P* = 0.001).

### 3.4 Autism and psychotic-like experiences

There were 12 studies examining the relationship between Autism and PLEs. Their characteristics and findings are presented in Table 2. From the total studies, seven reported a positive and significant correlation between autistic traits and PLE scores. In contrast, two studies reported that the association between autistic traits and PLE scores was positive but not significant. Three studies reported odds ratios indicating that individuals with higher autistic traits were more likely to experience PLEs. All three longitudinal studies reported that autistic traits significantly predicted PLEs. Similarly, all three studies involving individuals with a diagnosis of Autism reported that they were more likely to experience PLEs than those without. Six studies examined potential mediators and other relevant factors, including childhood trauma, social cognition and mentalizing biases. Importantly, two studies did not find a significant association between autistic traits and PLE scores.

**Table 2:** Autism and psychotic-like experiences: characteristics and findings of studies meeting inclusion criteria (*n* = 12).

**Author (year)**	**Country**	**Study design sample (number and diagnosis)**	**Mean age (SD) gender ratio (M/F)**	**Outcome measures**	**Main findings and clinical implications**
Abu-Akel et al. (2015)	UK	Cross-sectional 201 non-clinical	21.37 (SD = 4.32) 43 M/158 F	Community Assessment of Psychic Experiences (CAPE) Autism Quotient (AQ)	Autism and PLE scores were modestly but significantly associated (*r* = 0.31, *P* = 0.001). The interaction between AQ and CAPE scores was associated with a decrease in perspective taking errors (β = −0.002, *P* = 0.03). These findings provide support for the diametrical model of Autism and psychosis, in which their influences counteract the other (creating a “normality effect”).
Dardani et al. (2023)	UK	Longitudinal 13,105 non-clinical	Not reported 51% M/49% F	Psychosis-Like Symptoms interview (PLIKSi) Social Communication Disorder Checklist (SCDC)	Individuals with higher autistic traits were more likely to experience PLEs (OR = 1.13, *P* = 0.03). Social communication difficulties were also associated with PLEs (OR = 1.43, *P* = 0.04). Childhood trauma appeared to mediate the relationship between autistic traits and PLEs (NIE OR = 1.06, *P* = 0.001). These findings suggest that the relationship between Autism and PLEs may be influenced by environmental factors, rather than being entirely genetic. Additionally, early trauma work among autistic individuals could reduce risk of developing psychosis. This supports the “increased vulnerability” model of Autism and psychosis.
Hajdúk et al. (2023)	Slovakia	Cross-sectional 649 non-clinical	40.23 (SD = 13.09) 49% M/51% F	Community Assessment of Psychic Experiences (CAPE) The Comprehensive Autistic Trait Inventory	Exploratory graph analysis revealed associations between autistic symptoms and positive and negative PLEs. Difficulties in social relationships was strongly connected to negative PLE nodes. These findings indicate an overlap particularly between negative symptoms and autistic traits.
Jutla et al. (2022)	USA	Longitudinal 151 Autism 8,976 non-clinical	9.91 (SD = 0.62) 52% M/48% F	Prodromal Questionnaire—Brief Child version (PQ-BC) Autism based on report of diagnosis	Autistic individuals had higher PLE scores than those without: *t*(153) = −3.95, *P* = 0.001. Autism had a strong effect on PLE scores (β = 2.47, *P* = 0.000) and autistic individuals were more likely to have high PLE scores than those without (OR = 3.18, *P* = 0.000). These findings suggest a strong association between Autism and PLEs, in which autistic individuals are more likely to experience PLEs. These findings may also support the “increased vulnerability” model in which Autism increases risk to PLEs or psychosis.
Kreis et al. (2023)	Norway	Cross-sectional 52 non-clinical	23.50 (SD = 4.13) 21 M/31 F	Community Assessment of Psychic Experiences (CAPE) Autism Quotient (AQ)	AQ and CAPE scores were positively but not significantly correlated (ρ = 0.25, *P* = 0.08). In individuals with higher AQ scores, pupil dilation was less responsive to Bayesian surprise in volatile conditions but more responsive in cued task blocks (β = 0.05, *P* = 0.01). For individuals with higher CAPE scores, pupil dilation was less responsive to choice uncertainty in volatile conditions but more responsive in cued task blocks (β = 0.04, *P* = 0.03). These findings do not support a significant association between Autism and PLEs. However, they suggest that Autism and PLEs have different patterns of pupil dilation in response to cognitive and emotional factors, depending on the stability or predictability of the task environment.
Lisøy et al. (2022a)	Norway	Cross-sectional 321 non-clinical	32 (SD = 12.4) 103 M/179 F	Community Assessment of Psychic Experiences scale (CAPE) Autism Spectrum Quotient (AQ)	Autistic traits and PLEs were not significantly associated with preferred levels of complexity (*P* > 0.1). Similarly, autistic traits and PLEs were not significantly correlated (*P* > 0.05). Despite expecting to find an association between Autism, PLEs and preferred predictability, the findings were not significant. They also do not provide support for an association between Autism and PLEs.
Lisøy et al. (2022b)	Norway	Cross-sectional 26 with psychosis 300 non-clinical	31.60 (SD = 12.30) 45% M/54% F	Community Assessment of Psychic Experiences (CAPE) Autism Quotient (AQ)	There was a significant correlation (τ = 0.41) between autistic traits and PLEs. PLEs were significantly associated with hypermentalising (τ = −0.145), however autistic traits were not associated with hypomentalising. These findings did not support a diametrically opposite mentalizing bias between Autism and psychosis as hypothesized, however they do suggest an association between PLEs and hypermentalising. Additionally, negative symptoms were associated with hypomentalising which may link Autism and psychosis. While these findings do not provide support for the diametrical model, it is important to note that they do not refute it.
Mamah et al. (2021)	Kenya	Cross-sectional 9,564 non-clinical	21.2 (SD = 2.0) 53% M/47% F	Washington Early Recognition Center Affectivity and Psychosis (pWERCAP) Autism Spectrum Quotient (AQ)	There was a significant association between autistic traits and PLEs (*r* = 0.19, *P* < 0.001). These findings provide modest support for the link between Autism and PLEs, while broadening the range of evidence cross-culturally.
Martinez et al. (2021)	UK	Cross-sectional 14,853 non-clinical	Not reported	Psychosis Screening Questionnaire (PSQ) Autism Quotient (AQ)	Autistic traits and PLEs were significantly associated (β = 0.35, *P* = 0.001). Individuals with higher autistic traits were more likely to report PLEs (OR = 1.42, *P* = 0.001). Significant associations between autistic traits and paranoia (OR = 1.23), thought insertion (OR = 1.26) and strange experiences (OR = 1.22). These findings suggest that autistic traits may contribute to a vulnerability to PLEs. The significant associations with paranoia, thought insertion, and strange experiences suggest that individuals with higher autistic traits are not only more likely to experience PLEs in general but also specific types of psychotic symptoms.
Sijtsma et al. (2021)	Holland	Longitudinal 305 non-clinical	12.6 (SD = 0.4) 147 M/158 F	Diagnostic Interview for Children (DISC-C) Autism Symptom SElf-ReporT for Adolescents and Adults (ASSERT)	Autistic traits significantly predicted PLEs [*t*(289) = 3.2, *P* = 0.01]. There was no significant effect of autistic traits and PLEs on social cognition tasks. These findings highlight the strong relationship between autistic traits and PLEs, possibly indicating shared etiological mechanisms. Additionally, they provide support for the Increased Vulnerability model. The lack of a significant effect on social cognition tasks suggests that the mechanisms linking autistic traits to PLEs might be distinct from those underlying social cognitive abilities. Additionally, these findings do not provide support for the diametric model.
Suen et al. (2024)	Hong Kong	Cross-sectional 291 Autism 231 ADHD 59 co-occurring conditions 1,605 HC	19.76 (SD = 2.81) 42% M/58% F Ethnicity: Not reported	Autism-Spectrum Quotient (AQ-10) Generalized Anxiety Disorder (GAD-7) Prodromal Questionnaire Brief (PQ-B)	Individuals with co-occurring Autism and ADHD had significantly higher PLEs than the control group (mean difference = 5.23, *P* = 0.05). However, the difference between the mean scores of Autism alone, ADHD alone and the control group were non-significant. These findings suggest that the combination of Autism and ADHD represents a distinct and higher risk profile for PLEs compared to having either condition alone or being part of the general population.
van der Linden et al. (2020)	Holland	Cross-sectional 50 Autism 51 HC	41.1 (SD = 12.9) 35.5 (SD = 12.2) 26 M/24 F 26 M/25 F	Community Assessment of Psychic Experiences (CAPE) Momentary Psychotic Experiences (ESM)	Autism was strongly associated with increased PLEs (β = 0.49, *P* = 0.001). The strongest association was between PLEs and social stress in Autism (β = 1.21, *P* = 0.001). Individuals with Autism reported a higher total frequency of PLEs compared to controls (*P* = 0.001). Autistic individuals experienced greater overall distress from PLEs compared to controls (*P* = 0.000). This suggests that Autism is associated with a higher burden of PLEs in both their occurrence and the distress they cause, providing further support for the association between Autism and PLEs.

### 3.5 Autism and anxiety

There were 14 systematic reviews and meta-analyses exploring the relationship between Autism and anxiety. Their characteristics and findings are presented in Table 3. Of the included reviews, seven reported prevalence rates of anxiety disorders among individuals with an Autism diagnosis, with heterogeneous estimates possibly reflecting the varied populations including differences in age and IQ. Nine studies examined potential mediators and important factors involved in the relationship, including IU and sensory over-reactivity (SOR). Additionally, four of these reviews explored the relationship between IQ and anxiety in autistic individuals, with three concluding that higher IQ was associated with increased anxiety.

**Table 3:** Anxiety and autism: characteristics and findings of reviews meeting inclusion criteria (*n* = 14).

**Author (year)**	**Databases searched**	**Number of studies; type of review**	**Sample sizes in the included studies (total or range)**	**Population characteristics**	**Quality assessment tool used**	**Main findings and clinical implications**
Adams et al. (2023)	CINAHL, ERIC and PsycINFO	20; Meta-analysis	2,321	Participants were children and adolescents with an Autism diagnosis.	STROBE checklist	There was a negative relationship between anxiety and social competence in autistic young people, with higher anxiety being associated with lower social competence. The calculated effect sizes from the 22 independent samples ranged from −1.18 to 0.97, with a mean effect size of −0.48 (SD = 0.53). The relationship between anxiety and social competence was moderated by age, becoming weaker as age increased, suggesting that younger autistic individuals may be more negatively affected by anxiety in terms of their social competence.
Ambrose et al. (2021)	CINAHL, PsycINFO and ERIC	50; Systematic review	20 to 200	Participants were children and adolescents with an Autism diagnosis.	STROBE checklist	The review found clear associations between anxiety and poorer social relationships and increased victimization among autistic children. Children with elevated anxiety experienced more negative social experiences compared to those without anxiety. These findings indicate that the experiences of autistic individuals with high levels of anxiety may be poorer than those without anxiety.
Bougeard et al. (2021)	PubMed and Embase	46; Systematic review	936 to 631,619	Participants were children and adolescents with an Autism diagnosis.	Not reported	The included studies reported a wide range of rates of co-occurrence between Autism and anxiety, ranging from 0.00% to 82.20%. One study identified an IQ over 70 as a significant risk factor for increased anxiety in Autism (OR = 2.90), suggesting that higher cognitive abilities may contribute to greater anxiety.
Edirisooriya et al. (2021)	MEDLINE, PsycINFO and Scopus	15; Meta-analysis	1,049	Participants were children and adolescents with an Autism diagnosis.	Novel 5-point quality assessment score	The main analysis did not find a significant association between IQ and anxiety among autistic individuals. However, when only self-reported anxiety was considered, anxiety was found to be moderately higher among autistic individuals with lower IQ (*r* = −0.42, *P* = 0.01). These findings hint at a relationship between IQ and anxiety in Autism, however previous studies have found associations between both high and low IQ and anxiety. Self-report measures may reveal aspects of anxiety not captured by other methods.
Hollocks et al. (2019)	PsycINFO, PubMed and Web of Science	35; Systematic review and meta-analysis	26,070	Participants were children, adolescents and adults with an Autism diagnosis.	Assessed based on selection bias and detection bias	The included studies reported pooled estimates of current and lifetime prevalence for an anxiety disorder among autistic adults that ranged from 27 to 42%. These findings suggest that autistic adults are at increased risk of developing an anxiety disorder.
Jenkinson et al. (2020)	Scopus, Web of Science, PsycINFO and MEDLINE	12; Systematic review and meta-analysis	656	Participants were children, adolescents and adults with an Autism diagnosis or healthy controls.	Quality appraisal checklist for correlational studies (NICE, 2012)	Meta-analysis revealed elevated levels of anxiety and intolerance of uncertainty among autistic individuals compared to controls. Anxiety and intolerance of uncertainty were significantly correlated among autistic individuals when effect sizes were pooled (*r* = 0.62, *P* = 0.001). These findings identify intolerance of uncertainty as a key component of anxiety in Autism and may offer a target for intervention to reduce anxiety disorders in Autism.
Kilanko et al. (2022)	Embase, PubMed and Google Scholar	5; Systematic review	122 to 221,694	Participants were children, adolescents and adults with an Autism diagnosis.	Not reported	The included studies reported a wide range of factors that may influence anxiety in Autism, such as gender and verbal IQ. One included study found women to be at greater risk of anxiety than men. Another reported that autistic children with higher functioning experienced greater levels of anxiety. These findings demonstrate how a variety of factors may influence rates of anxiety among autistic individuals.
Lai et al. (2019)	MEDLINE, Embase, PsycINFO, Scopus and Web of Science	100; Systematic review and meta-analysis	53,243 to 210,249	Participants were children, adolescents and adults with an Autism diagnosis.	Hoy Risk of Bias Tool	Meta-analysis revealed an overall pooled prevalence estimate of 20% for anxiety disorders among autistic individuals. These findings support the elevated rate of anxiety disorders among autistic individuals and demonstrate how several factors may influence this.
Micai et al. (2023)	PubMed and PsycINFO	340; Systematic review and meta-analysis	590,000	Participants were children, adolescents and adults with an Autism diagnosis.	Hoy Risk of Bias Tool	The included studies revealed that anxiety disorders were one of the most prevalent co-occurring conditions in Autism, with a pooled estimate of 35%. These findings demonstrate the increased rate of anxiety disorders among autistic individuals.
Mingins et al. (2021)	PsycINFO, PsycARTICLES and MEDLINE	49; Systematic review and meta-analysis	18,430	Participants were children and adolescents with an Autism diagnosis.	Quality Assessment Framework table	The meta-analysis revealed a significant correlation between IQ and anxiety levels in autistic children. Specifically, studies that included children with intellectual disability showed a moderate effect size (*r* = 0.18), indicating that higher anxiety levels were associated with higher IQ scores. In group-level analyses, no significant difference in anxiety scores was found between children with intellectual disability and those without intellectual disability. These findings suggest that the relationship between anxiety and IQ may not be straightforward, however there appears to be an association.
Montaser et al. (2023)	PubMed, Medline and ScienceDirect	10; Systematic review	97 to 194	Participants were individuals diagnosed with social anxiety.	Newcastle-Ottawa Scale (NOS), Scale for the Assessment of Narrative Review Articles (SANRA)	The included studies support the theory that social anxiety in Autism differs from that in neurotypical individuals. The included prevalence studies reported higher levels of social anxiety among autistic individuals. Different underlying mechanisms may distinguish the two, such as greater intolerance of uncertainty as well as genuine difficulties in social interactions which are viewed negatively by others. These findings suggest that autistic individuals may be at greater risk of social anxiety. Additionally, while neurotypical individuals with social anxiety may perceive themselves more negatively than others do, autistic individuals may genuinely struggle socially which is noticeable to others.
Mutluer et al. (2022)	PubMed and Web of Science	29; Meta-analysis	7,791 to 133,233	Participants were children and adolescents with an Autism diagnosis.	Egger's test for publication bias	Meta-analysis revealed a pooled prevalence of 11.1% for anxiety disorders among autistic individuals. This offers further support for a significant rate of anxiety disorders among autistic individuals.
van Steensel et al. (2011)	PsyInfo, Pubmed, Web of Science and ERIC	31; Meta-analysis	2,121	Participants were children and adolescents with an Autism diagnosis.	Egger's test for publication bias	The meta-analysis found that 39.6% of autistic individuals had at least one co-occurring anxiety disorder. The most common were specific phobia (29.8%), OCD (17.4%) and social anxiety disorder (16.6%). These findings demonstrate the increased rate of anxiety disorders among autistic individuals, as well as suggesting that certain anxiety disorders may be more common. Furthermore, the symptom overlap between OCD, social anxiety and Autism may contribute to the greater prevalence.
Vasa et al. (2020)	PubMed, PsycINFO, the Cochrane Library and Embase	44; Systematic review	11 to 2,114	Participants were children and adolescents with an Autism diagnosis.	Not reported	The prevalence of anxiety in young children across the included studies ranged from 1.6 to 62%. Factors such as cognitive and social functioning, along with sensory over-responsivity, were linked to anxiety. Additionally, three longitudinal studies observed a rise in anxiety levels over time. These findings highlight the wide range of factors that may influence the prevalence of anxiety disorders among autistic individuals.

## 4 Discussion

This systematic review aimed to synthesize the literature examining whether anxiety increases vulnerability to PLEs in autistic individuals. Due to the lack of research directly exploring this topic, three separate searches were conducted to address the research question. Higher levels of anxiety were associated with increased frequency of PLEs, and there was evidence supporting a bidirectional relationship between the two. Additionally, autistic traits were linked to higher rates of PLEs, and autistic individuals were more likely to experience PLEs compared to healthy controls. Finally, an Autism diagnosis was consistently associated with increased rates of anxiety disorders.

### 4.1 Anxiety and psychotic-like experiences

A consistent finding of the current review was the association between anxiety scores and increased rates of PLEs within nonclinical populations, indicating that in individuals without a known Autism diagnosis, higher anxiety levels correlated with more frequent PLEs. There appears to be only one previous systematic review on this topic, focused on children and adolescents, which similarly reported evidence of a strong relationship between anxiety and PLEs that highlights the causal role of affective distress in the development of psychotic symptoms (Bird et al., 2018). Notably, the review reported that the strongest association was between anxiety and paranoia, a finding echoed by one of the studies included in the current review. These observations align with the theoretical model proposed by Freeman (2007), which posits that anxiety is central to the development of delusions and paranoid ideas. The model is supported by prior research linking anxiety, paranoia and persecutory beliefs suggesting that anxiety may create a state of heightened threat perception which increases vulnerability to misinterpreting anomalous experiences in the form of PLEs (Huppert and Smith, 2005; Wigman et al., 2012).

Another key finding was longitudinal evidence for a bidirectional relationship between anxiety and PLEs, where each increases the likelihood of developing the other. This is consistent with previous research highlighting the distress and anxiety caused by experiencing PLEs, suggesting a cyclical relationship in which anxiety and PLEs reinforce each other (Brett et al., 2014; Armando et al., 2010). Interestingly, the predictive link between anxiety and PLEs may reflect shared vulnerabilities, as research has identified common underlying neurobiological mechanisms in both conditions (Varghese et al., 2011). For instance, HPA dysregulation is consistently implicated in the relationship between anxiety and psychotic symptoms, with preliminary research suggesting a similar association with PLEs (Phillips et al., 2006; Walker et al., 2008). In addition, reduced cortical thickness in the temporal lobe has been linked with each condition, suggesting that structural brain abnormalities could underlie the susceptibility to both anxiety and PLEs (Vargas and Mittal, 2023; Zink et al., 2008). These findings emphasize the neurobiological similarities between PLEs and anxiety disorders, offering insight into their relationship when considered separately from Autism.

Furthermore, there is evidence to suggest that anxiety and PLEs may share similar underlying cognitive mechanisms. One example is the cognitive bias “jumping to conclusions”, in which individuals make hasty decisions based on limited evidence (So et al., 2008; Dudley et al., 2016). This bias is thought to mediate the pathway between anxiety and paranoia, as anxious individuals may be more prone to making quick, unfounded judgments that can lead to paranoid thinking (Lincoln et al., 2010). Similarly, one study in the current review reported that “external attribution” bias significantly predicted PLEs among participants with high anxiety (Prochwicz and Kłosowska, 2018). This reasoning bias is often implicated in the development of delusions, and therefore may reflect another important mechanism linking anxiety and PLEs (Janssen et al., 2006). Further research establishing the role of cognitive processes in this relationship could allow for the development of interventions addressing these faulty cognitions.

### 4.2 Autism and psychotic-like experiences

Autistic traits were consistently associated with increased frequency of PLEs in this review: five of the included studies reported significant associations within nonclinical samples, while two compared autistic participants to healthy controls. These findings are consistent with the only previous systematic review examining the topic, which similarly reported an increased rate of PLEs among autistic individuals (Kiyono et al., 2020). However, it is important to note that two studies in the current review did not find a significant association between the two variables. This may be explained in two ways: firstly, Kreis et al. (2023) only included 52 participants in their study, which may have been inadequate to detect a meaningful effect. Since both Autism and psychosis occur in approximately 1% of the population, research involving less than 100 participants may lack the statistical power to detect significant associations (Chisholm et al., 2015). Although the second study involved a suitable number of participants (*n* = 321), the heterogeneity in definitions and outcome measures used for both PLEs and autistic traits may account for these conflicting findings.

Within the wider literature, Autism is most frequently associated with the negative symptoms of psychosis, such as social withdrawal and flat affect (Trevisan et al., 2020). Central features of Autism, such as deficits in theory of mind and face processing, have been recognized in individuals with psychosis and may be linked with the development of negative symptoms (Catalan et al., 2018; Darke et al., 2021). This is a key distinction between Autism and anxiety, which is more often associated with positive symptoms, such as hallucinations and delusions (Huppert and Smith, 2005). However, the current review similarly found stronger evidence linking autistic traits with positive PLEs, contrasting broader psychosis research. Perhaps this may reflect subtle differences between PLEs and psychosis, or more likely, it could be due to several included studies focusing solely on the positive subscale of the CAPE questionnaire. While positive PLEs are more directly linked to the risk of developing psychosis, future research should explore the association between negative PLEs and Autism to determine whether it mirrors the established relationship between Autism and the negative symptoms of psychosis (Cowan and Mittal, 2021).

Drawing on theoretical models, IU is frequently identified as a causative factor in both conditions (Lisøy et al., 2022b). Interestingly, a recent study hypothesized that cognitive disturbances such as IU, combined with affective distress such as anxiety, may result in a faulty appraisal process which leads to the development of PLEs (Armando, 2023). This difficulty in coping with ambiguous situations may therefore link anxiety, Autism and PLEs. In addition, one study included in the current review found evidence of diminished pupil responses in both participants with higher autistic traits and those experiencing frequent PLEs. This finding supports previous research that identified aberrant norepinephrinergic signaling, indicated by diminished pupil response, as a shared mechanism underlying both Autism and psychosis (Thakkar et al., 2018; Zhao et al., 2022). Norepinephrinergic signaling plays a crucial role in stress response, and abnormalities in this system may contribute to IU in both conditions and subsequently the development of PLEs (Lawson et al., 2017; Kreis et al., 2023, 2022).

Furthermore, while anxiety and PLEs may share similar cognitive styles, research indicates that those characterizing Autism and PLEs may differ (Fernandes et al., 2018). Three of the studies included in the current review explored the cognitive processes involved in this relationship, and similarly identified important distinctions (Abu-Akel et al., 2015; Lisøy et al., 2022a; Sijtsma et al., 2021). Autistic traits and PLEs were found to counterbalance each other on a number of perspective-taking tasks, suggesting that the presence of higher autistic traits might have an attenuating effect on the cognitive errors associated with psychosis proneness (Abu-Akel et al., 2015). This aligns with previous literature asserting that cognitions in Autism may be more deliberate and methodical than the “jumping to conclusions” cognitive style often seen in anxiety and psychosis (Brosnan et al., 2014). In addition, PLEs were significantly associated with hypermentalizing, which highlights another potential cognitive mechanism involved in their development (Lisøy et al., 2022a). While the study did not find a contrasting link between autistic traits and hypomentalizing as anticipated, this may reflect the study's focus on subclinical autistic traits rather than individuals with an Autism diagnosis, who may display more pronounced impairments in mentalization (Chung et al., 2014).

Moreover, anxiety may influence the relationship between Autism and PLEs through the increased levels of distress experienced by autistic individuals in response to PLEs: one of the included studies reported that autistic individuals experienced heightened anxiety in reaction to PLEs, which served to increase both the frequency and the distress associated with these experiences (van der Linden et al., 2020). In addition, two studies highlighted the influence of shared environmental factors in explaining the relationship between Autism and PLEs. Experiences such as bullying and victimization, to which autistic individuals may be more vulnerable, may increase the risk of developing psychotic symptoms later in life (Sijtsma et al., 2021; Bortoletto et al., 2023). Similarly, childhood trauma was identified as a potential mediator in the relationship, offering further support for the role of environmental factors in the increased rates of PLEs seen in Autism (Dardani et al., 2023). Since the aforementioned environmental factors are commonly associated with high levels of stress and anxiety (Gong et al., 2022), these findings may also provide indirect evidence for the causal role of anxiety in PLEs in Autism.

Additionally, previous research has identified substantial neurobiological and genetic overlap between Autism and psychosis, which may elucidate the association between autistic traits and PLEs (de Lacy and King, 2013). A consistent finding is that Autism and psychosis appear to share similar white matter deficits, whilst displaying diametrically opposite patterns of gray matter volumetry (Mitelman et al., 2017). Specifically, Autism has been linked with increased gray matter volume, while reduced gray matter volume has been identified in psychosis (Mitelman et al., 2018). This may reflect the complex association between Autism and psychosis, in which similarities, such as difficulties in social interaction and flat affect, are juxtaposed by marked differences, including how sensory information is processed (Barlati et al., 2016). Furthermore, research has identified higher levels of oxidative biomarkers in both conditions that may be involved in the etiology of Autism and PLEs (Smaga et al., 2015). These findings, considered alongside evidence of shared environmental influences, indicate that stress and inflammation may be an important therapeutic target for preventing PLEs in autistic populations.

### 4.3 Autism and anxiety

Seven reviews found increased rates of anxiety disorders among autistic individuals, with pooled prevalence estimates ranging from 11 to 40% (Mutluer et al., 2022; van Steensel et al., 2011). Moreover, individual study estimates reported prevalence rates of as high as 80% (Salazar et al., 2015). These findings align with a previous umbrella review exploring common co-occurring conditions in Autism, which similarly found increased levels of anxiety in autistic individuals across the spectrum (Hossain et al., 2020). However, it is important to acknowledge the significant variability in the reported prevalence estimates, as this may introduce uncertainty when attempting to draw definitive conclusions. The discrepant findings of the included reviews likely reflect the heterogeneity of the participants involved, such as vast differences in age and ability, as well as the wide variety of methodologies used (Hobson and Petty, 2021). Nevertheless, the overall trend across the included reviews indicates a strong association between anxiety and Autism.

In addition, several of the included reviews examined potential factors involved in the relationship between anxiety and Autism. One meta-analysis reported a strong correlation between IU and anxiety among autistic participants (Jenkinson et al., 2020). This provides important support for the role of IU in the relationship between anxiety, Autism and PLEs, suggesting that heightened threat perception may be an important factor linking the three variables (South and Rodgers, 2017). Similarly, SOR was identified in one of the included reviews as a relevant mechanism involved in anxiety in Autism (Vasa et al., 2020). While SOR is not frequently associated with PLEs or psychosis, previous research has identified abnormalities in electrodermal response in both Autism and schizophrenia (Reynolds and Lane, 2008). This suggests that both autistic individuals and those with schizophrenia may have atypical ways of processing sensory information. In Autism, this may manifest as SOR, leading to heightened anxiety (Lydon et al., 2016); in schizophrenia, similar sensory processing issues may contribute to the sensory distortions or hallucinations that are characteristic of psychosis (Dawson and Schell, 2002).

Furthermore, three of the included reviews reported that higher IQ was associated with increased anxiety (Bougeard et al., 2021; Kilanko et al., 2022; Mingins et al., 2021), while one identified an association between lower IQ and anxiety (Edirisooriya et al., 2021). High IQ may be associated with a greater ability for abstract thinking and advanced cognitive processing, which would allow for greater worrying and internalization (Mingins et al., 2021). Conversely, individuals with lower IQ may be unable to develop coping strategies when dealing with unfamiliar situations, leading to increased levels of stress and anxiety (Edirisooriya et al., 2021). This may have important implications when considering the wider relationship between anxiety, Autism and PLEs, as it could highlight certain groups that are at greater risk of psychosis. Premorbid IQ deficits have been identified in individuals with schizophrenia, therefore exploring the role of IQ in PLEs and Autism may be a potential avenue for future research (Rajji et al., 2009).

### 4.4 Anxiety, autism, and psychotic-like experiences

Despite the lack of literature examining anxiety, Autism and PLEs, there is some research on anxiety disorders, Autism and psychosis, which can be used to further contextualize the findings of the current review. Studies conducted within adult populations often juxtapose individuals with social anxiety disorder (SAD), Autism and psychosis, as they are three prominent conditions characterized by social deficits (Chan et al., 2019; Pepper et al., 2019; Demetriou et al., 2020; Pepper et al., 2018). While this provides evidence of similarities in social functioning, it offers little perspective on the causal relationship between the three variables. Greater insight may instead be gained from previous research conducted among children: Kyriakopoulos et al. (2015) examined anxiety in 84 autistic children admitted to a specialist inpatient unit, finding that anxiety symptoms mediated the pathway between Autism and psychotic symptoms within this sample. Formal thought disorder, a syndrome associated with psychotic disorders, was highlighted as an important aspect that may be induced by anxiety in autistic children. This is corroborated by two previous case-control studies, in which anxiety scores were significantly associated with illogical thinking and loose associations in autistic children (Gaag et al., 2005; Solomon et al., 2008). Interestingly, this may implicate abnormal connectivity in certain brain structures, such as the frontotemporal network, in the relationship between anxiety, Autism and psychosis (Caplan et al., 2001). In addition, an earlier paper found that anxious children with pervasive developmental disorders, such as autism, presented with more severe psychotic symptoms (Weisbrot et al., 2005). Collectively, these findings provide preliminary evidence for the potential causal role of anxiety in the development of psychosis, however further research is needed in adult populations.

### 4.5 Limitations

This is the first review to synthesize literature exploring anxiety, Autism and PLEs, identifying an important area for future research. A key strength of this review is that the quality of the included studies was generally high (see Supplementary Tables 1–4). However, there are several limitations. Firstly, the lack of research directly examining anxiety, Autism and PLEs is a significant disadvantage, as it limits the extent to which the research question can be addressed beyond discussion of bilateral associations between the variables. Similarly, most research included in the current review was cross-sectional, which precludes any assessment of causality, particularly in relation to the causal role of anxiety; the limited number of longitudinal studies included in this review offer more robust insight into the temporal dynamics between anxiety and PLEs. Despite overall quality, clear effect was rated “Unclear” in 21 out of 31 cross-sectional studies (see Supplementary Table 1), meaning that any conclusions drawn must remain tentative. The huge heterogeneity of methods utilized in the included studies meant a meta-analysis was not possible, which would have strengthened the findings of this review. Furthermore, much of the included research involved measuring PLEs, autistic traits and anxiety scores within nonclinical populations. As the research question pertains to autistic individuals, it is unclear whether the included studies can be used to answer the research question or be applied to clinical populations.

A further limitation of the current review is the generalizability of the included studies, as many involved online questionnaires or student populations. While online questionnaires are both cost- and time-efficient, they may not capture samples that are reflective of the general population, including those who do not or cannot use computers (Gosling et al., 2010). Similarly, despite being easy to recruit, student populations are often socioeconomically and ethnically unrepresentative (Hanel and Vione, 2016). Studies conducted in broader populations frequently used sampling methods that involved contacting addresses in a certain region, excluding homeless populations and those in temporary accommodation, who may be at greater risk of PLEs (Byrne et al., 2015). Consequently, this may limit the generalizability and application of any conclusions drawn if the included studies are not representative. Finally, perhaps the most important limitation is that this review was conducted independently by one author. Collaboration is an important element of systematic reviews, and while initial screening was confirmed by an independent rater, decisions made at later stages may have benefited from a collaborator. Ultimately, bias may be inherent in the decisions made by one person.

### 4.6 Implications and future directions

This systematic review identified strong associations between anxiety, Autism and PLEs, suggesting that both anxiety symptoms and autistic traits are important factors in the emergence of PLEs. These findings have important implications for early intervention: firstly, they emphasize the importance of screening for PLEs in autistic individuals, as well as those with anxiety disorders, as this could lead to the early identification of individuals at greater risk of developing psychosis. Similarly, autistic individuals with a co-occurring anxiety disorder should be recognized as an at-risk group in mental health services, as they may benefit from individualized prevention strategies. For instance, interventions aimed at treating anxiety in autistic individuals may have the potential to reduce rates of transition to psychosis in this population. Additionally, the current review identified IU as an important mechanism in the relationship between the three variables, suggesting it may be a useful target for therapeutic intervention. Improving tolerance to uncertainty could benefit individuals at risk through reducing anxiety, and could inform relapse prevention work in autistic individuals who have already experienced a first episode of psychosis (Miller and McGuire, 2023). Other cognitive biases were proposed as important contributing factors, suggesting that Cognitive Behavioral Therapy may be especially useful in this population. Moreover, this review highlights the importance of addressing environmental influences, which may mediate the relationship between Autism and PLEs. Psychosocial interventions that address trauma and focus on reducing environmental stressors could therefore be effective in decreasing the incidence of PLEs in autistic individuals.

Nevertheless, further research is needed involving adult populations exploring anxiety, Autism and PLEs, which would allow for a future systematic review to be conducted involving all three search terms. For example, case-control studies could compare PLE frequency among autistic individuals and a co-occurring anxiety disorder to those without, providing important data for future meta-analysis. As availability of consistent datasets in the field increases, both standard and network meta-analyses would allow for more robust comparisons to be made. In particular, a network meta-analysis approach would enable researchers to account for the overlap between subclinical psychosis risk measures and diverse anxiety metrics, offering a more nuanced understanding of their interrelation and enhancing the precision of comparative analyses. It is important to note that OCD is no longer categorized as an anxiety disorder in the DSM-5, despite five of the included studies conceptualizing it as such (American Psychiatric Association, 2022). Previous research has highlighted a relationship between OCD and psychosis distinct from that of other anxiety disorders; therefore, this may have implications for the conclusions drawn from studies that have not accounted for this (Cunill et al., 2009; Martinho et al., 2023). As the field of research continues to develop, a systematic review specifically exploring the relationship between OCD, Autism and psychotic-like experiences may provide useful insight into the co-occurring conditions.

However, the current reliance on self-report methods may result in false estimations of PLE frequency, as both recall bias, and the abstract construct of PLEs, can make interpreting questions in a standardized way challenging (Kelleher and Cannon, 2011; Prochwicz and Gaweda, 2016). Despite this, interviews are thought to increase the chance of socially desirable answering in PLE research, due to the sensitive nature of the topic and the influence of mental health stigma (Kelleher and Cannon, 2011; Mamah et al., 2021). Instead, future research may benefit from utilizing observer-ratings in addition to self-report measures, in order to reduce bias and enhance methodological validity (Spitz et al., 2017). In addition, establishing standardized definitions of PLEs would allow for clearer comparison between studies (Hinterbuchinger and Mossaheb, 2021). Structural Equation Modeling (SEM) could be utilized to examine anxiety as a mediator in the relationship between autistic traits and PLEs (Gunzler et al., 2013). Future investigations should look deeper into the distress, insight and subjective quality of PLEs rather than simply the frequency, moving beyond prevalence estimates and taking a more phenomenological approach. Lastly, further longitudinal research is needed to disentangle the temporal and causal relationships between anxiety, PLEs, and autistic traits. Establishing the causal role of anxiety in the development of PLEs in Autism would be of significant benefit to the wider field of early intervention in psychosis (Bird et al., 2018).

## 5 Conclusions

This review has presented systematic evidence of the strong associations between anxiety, Autism and PLEs, identifying key mechanisms involved in these relationships. While providing useful insight into the etiology of PLEs in Autism, this review has also highlighted significant gaps in the literature, such as the lack of case-control studies and longitudinal research examining the causal role of anxiety. Future research addressing these gaps could guide the development of targeted psychological interventions, to reduce rates of psychosis transition and, ultimately, improve outcomes for autistic individuals.

## Data Availability

The original contributions presented in the study are included in the article/Supplementary material, further inquiries can be directed to the corresponding author.
