# Supplementary material for: The relationships between anxiety, psychotic-like experiences and autism: a systematic review
Source: Front Psychol. 2025 Oct 13;16:1549886. doi: 10.3389/fpsyg.2025.1549886 (PMC12554707; doi:10.3389/fpsyg.2025.1549886)
Supplement: Supplementary file 1 [file Supplementary_file_1.docx]

Supplementary Material

# Supplementary Tables

## Supplementary Table 1. CASP assessment – cross-sectional studies

| **Author** | **Clear focus?** | **Appropriate method?** | **Case recruitment acceptable?** | **Control recruitment acceptable?** | **Exposure measured accurately?** | **Groups treated equally?** | **Confounding factors considered?** | **Clear effect?** | **Precise estimate?** | **Believable results?** | **Applicable results?** | **Congruent results?** |
| --- | --- | --- | --- | --- | --- | --- | --- | --- | --- | --- | --- | --- |
| Abu-Akel et al. (2015) | Y | Y | U | N/A | Y | N/A | Y | U | Y | Y | Y | Y |
| Antosz-Rekucka & Prochwicz (2023) | Y | Y | Y | N/A | Y | N/A | N | U | Y | Y | Y | Y |
| Armando et al. (2013) | Y | Y | Y | Y | Y | Y | Y | U | Y | Y | Y | Y |
| Baryshnikov et al. (2018) | Y | Y | Y | N/A | Y | N/A | Y | U | Y | Y | Y | Y |
| Bortolon & Raffard (2015) | Y | Y | Y | Y | Y | Y | Y | U | Y | Y | Y | Y |
| Bourgin et al. (2020) | Y | Y | Y | N/A | Y | N/A | Y | Y | Y | Y | Y | Y |
| Byrne, Steele & Pachana (2015) | Y | Y | Y | N/A | N | N/A | Y | Y | Y | Y | Y | Y |
| Deng, Grove & Deldin (2020) | Y | Y | Y | N/A | Y | N/A | Y | U | Y | Y | Y | Y |
| Ered et al. (2018) | Y | Y | Y | N/A | Y | N/A | Y | U | Y | Y | Y | Y |
| Freeman & Fowler (2009) | Y | Y | Y | N/A | Y | N/A | Y | U | Y | Y | Y | Y |
| Hajdúk et al. (2023) | Y | Y | Y | N/A | Y | N/A | N | U | Y | Y | Y | Y |
| Kreis et al. (2023) | Y | Y | Y | N/A | Y | N/A | Y | U | Y | Y | U | N |
| Lisøy et al. (2022a) | Y | Y | Y | N/A | Y | N/A | Y | U | Y | Y | Y | N |
| Lisøy et al. (2022b) | Y | Y | Y | Y | Y | Y | Y | U | Y | Y | Y | U |
| Lu et al. (2022) | Y | Y | Y | N/A | Y | N/A | Y | Y | Y | Y | Y | Y |
| Mamah et al. (2021) | Y | Y | Y | N/A | Y | N/A | N | U | Y | Y | Y | Y |
| Martinez et al. (2021) | Y | Y | Y | N/A | Y | N/A | Y | Y | Y | Y | Y | Y |
| Merola et al. (2023) | Y | Y | Y | Y | Y | Y | Y | U | Y | Y | Y | U |
| Monsonet et al. (2022) | Y | Y | Y | Y | Y | Y | Y | U | Y | Y | Y | Y |
| Park et al. (2022) | Y | Y | Y | Y | Y | Y | Y | U | Y | Y | Y | Y |
| Prochwicz & Gawęda (2016) | Y | Y | Y | N/A | Y | N/A | Y | U | Y | Y | Y | Y |
| Prochwicz & Kłosowska (2018) | Y | Y | Y | Y | Y | Y | Y | Y | Y | Y | Y | Y |
| Rejek et al. (2023) | Y | Y | Y | N/A | Y | N/A | Y | U | Y | Y | U | Y |
| Rep et al. (2023) | Y | Y | Y | N/A | Y | N/A | Y | Y | Y | Y | Y | Y |
| Saha et al. (2012) | Y | Y | Y | N/A | Y | N/A | Y | Y | Y | Y | Y | Y |
| Suen et al. (2024) | Y | Y | Y | Y | Y | Y | Y | U | Y | Y | Y | Y |
| Unterrassner et al. (2017) | Y | Y | Y | N/A | Y | N/A | Y | U | Y | Y | Y | Y |
| van der Linden et al. (2020) | Y | Y | Y | Y | Y | Y | Y | U | Y | Y | Y | Y |
| Xu, Chen & Wang (2024) | Y | Y | Y | N/A | Y | N/A | Y | Y | Y | Y | Y | Y |
| Yang et al. (2023) | Y | Y | Y | N/A | Y | N/A | Y | Y | Y | Y | Y | Y |
| Yilmaz Kafali et al. (2022) | Y | Y | Y | Y | Y | Y | Y | Y | Y | Y | Y | Y |

# Y – Yes, N – No, U – Unclear

## Supplementary Table 2. CASP assessment – longitudinal studies

| **Author** | **Clear focus?** | **Cohort recruitment acceptable?** | **Exposure measured accurately?** | **Outcome measured accurately?** | **Confounding factors considered?** | **Confounding factors accounted for?** | **Follow-up complete enough?** | **Follow-up long enough?** | **Clear results?** | **Precise results?** | **Believable results?** | **Applicable results?** | **Congruent results?** |
| --- | --- | --- | --- | --- | --- | --- | --- | --- | --- | --- | --- | --- | --- |
| Dardani et al. (2023) | Y | Y | Y | Y | Y | Y | Y | Y | Y | Y | Y | Y | Y |
| Giocondo et al. (2021) | Y | Y | Y | Y | Y | U | Y | Y | Y | Y | Y | Y | Y |
| Isaksson et al. (2020) | Y | Y | Y | Y | Y | Y | Y | Y | Y | Y | Y | Y | Y |
| Isaksson et al. (2022) | Y | Y | Y | Y | Y | N | Y | Y | Y | Y | Y | Y | Y |
| Jutla et al. (2022) | Y | Y | Y | Y | Y | Y | N | Y | Y | Y | Y | Y | Y |
| Morales-Muñoz et al. (2022) | Y | Y | Y | Y | Y | N | N | Y | Y | Y | Y | Y | Y |
| Sijtsma et al. (2021) | Y | Y | Y | Y | Y | Y | N | Y | Y | Y | Y | Y | Y |
| Varghese et al. (2011) | Y | Y | Y | Y | Y | Y | Y | Y | Y | Y | Y | Y | Y |
| Wu et al. (2021) | Y | Y | Y | Y | Y | U | Y | Y | Y | N | Y | Y | Y |
| Yamasaki et al. (2018) | Y | Y | Y | Y | Y | N | Y | Y | Y | Y | Y | Y | Y |

# Y – Yes, N – No, U – Unclear

## Supplementary Table 3. CASP assessment – systematic reviews

| **Author** | **Clear focus?** | **Acceptable search strategy?** | **Relevant studies included?** | **Quality assessed?** | **Results combined effectively?** | **Clear results?** | **Precise results?** | **Applicable results?** | **Important outcomes considered?** | **Benefits worth costs?** |
| --- | --- | --- | --- | --- | --- | --- | --- | --- | --- | --- |
| Ambrose, Simpson & Adams (2021) | Y | Y | Y | Y | Y | Y | N | Y | Y | Y |
| Bougeard et al. (2021) | Y | Y | Y | N | Y | Y | Y | Y | Y | Y |
| Kilanko et al. (2022) | Y | Y | N | N | Y | Y | N | Y | Y | Y |
| Micai et al. (2023) | Y | Y | Y | Y | Y | Y | Y | Y | Y | Y |
| Montaser et al. (2023) | Y | Y | Y | Y | Y | Y | N | Y | Y | Y |
| Vasa et al. (2020) | Y | Y | Y | N | Y | Y | Y | Y | Y | Y |

# Y – Yes, N – No, U – Unclear

## Supplementary Table 4. CASP assessment – systematic review and meta-analyses

| **Author** | **Clear focus?** | **Acceptable search strategy?** | **Relevant studies included?** | **Validity/rigor assessed?** | **Appropriate presentation?** | **Appropriate analysis?** | **Limitations reported clearly?** | **Applicable results?** | **Benefits worth costs?** | **Does it add value?** |
| --- | --- | --- | --- | --- | --- | --- | --- | --- | --- | --- |
| Adams et al. (2023) | Y | Y | Y | Y | Y | Y | Y | Y | Y | Y |
| Edirisooriya et al. (2021) | Y | Y | Y | Y | Y | Y | Y | Y | Y | Y |
| Hollocks et al. (2019) | Y | Y | Y | Y | Y | Y | Y | Y | Y | Y |
| Jenkinson & Thompson (2020) | Y | Y | Y | Y | Y | Y | Y | Y | Y | Y |
| Lai et al. (2019) | Y | Y | Y | Y | Y | Y | Y | Y | Y | Y |
| Mingins et al. (2021) | Y | Y | Y | Y | Y | Y | Y | Y | Y | Y |
| Mutluer et al. (2022) | Y | Y | Y | Y | Y | Y | Y | Y | Y | Y |
| van Steensel, Bogels & Perrin (2011) | Y | Y | Y | Y | Y | Y | Y | Y | Y | Y |

# Y – Yes, N – No, U – Unclear

# Supplementary Material

**2.2. Supplementary Material 1.** Screening and Data Extraction

Studies that appeared to be eligible were retrieved for full-text assessment, conducted by one author and confirmed by an independent rater. This was another student from the same MSc course who independently conducted a systematic search using the same key words to identify eligible studies. Once both parties had completed Data Extraction tables, these were cross-examined to confirm that the same studies had been retrieved. Any uncertainty regarding study eligibility was resolved through discussion with a supervising author to reach consensus.
